# Attachment style predicts affect, cognitive appraisals, and social functioning in daily life

**DOI:** 10.3389/fpsyg.2015.00296

**Published:** 2015-03-18

**Authors:** Tamara Sheinbaum, Thomas R. Kwapil, Sergi Ballespí, Mercè Mitjavila, Charlotte A. Chun, Paul J. Silvia, Neus Barrantes-Vidal

**Affiliations:** ^1^Departament de Psicologia Clínica i de la Salut, Facultat de Psicologia, Universitat Autònoma de BarcelonaBarcelona, Spain; ^2^Department of Psychology, University of North Carolina at Greensboro, Greensboro, NCUSA; ^3^Sant Pere Claver – Fundació SanitàriaBarcelona, Spain; ^4^Centre for Biomedical Research Network on Mental Health, Instituto de Salud Carlos III, MadridSpain; ^5^Red de Excelencia PROMOSAM (PSI2014-56303-REDT), MINECOSpain

**Keywords:** adult attachment, Attachment Style Interview, experience sampling, ecological validity, individual differences

## Abstract

The way in which attachment styles are expressed in the moment as individuals navigate their real-life settings has remained an area largely untapped by attachment research. The present study examined how adult attachment styles are expressed in daily life using experience sampling methodology (ESM) in a sample of 206 Spanish young adults. Participants were administered the Attachment Style Interview (ASI) and received personal digital assistants that signaled them randomly eight times per day for 1 week to complete questionnaires about their current experiences and social context. As hypothesized, participants’ momentary affective states, cognitive appraisals, and social functioning varied in meaningful ways as a function of their attachment style. Individuals with an anxious attachment, as compared with securely attached individuals, endorsed experiences that were congruent with hyperactivating tendencies, such as higher negative affect, stress, and perceived social rejection. By contrast, individuals with an avoidant attachment, relative to individuals with a secure attachment, endorsed experiences that were consistent with deactivating tendencies, such as decreased positive states and a decreased desire to be with others when alone. Furthermore, the expression of attachment styles in social contexts was shown to be dependent upon the subjective appraisal of the closeness of social contacts, and not merely upon the presence of social interactions. The findings support the ecological validity of the ASI and the person-by-situation character of attachment theory. Moreover, they highlight the utility of ESM for investigating how the predictions derived from attachment theory play out in the natural flow of real life.

## Introduction

Attachment theory (Bowlby, 1973, 1980, 1982), along with its theoretical and empirical extensions (e.g., Main, 1990; Schore, 1994; Mikulincer and Shaver, 2003), is a useful and influential framework for understanding personality development, relational processes, and the regulation of affect. Over the past two decades, an increasing body of research has accrued on the origins and correlates of individual differences in adult attachment styles (Mikulincer and Shaver, 2007). However, an important limitation of previous studies is that many failed to take into account the effect of context on the expression of attachment styles. This is surprising given that attachment theory is in essence a “person by situation” interactionist theoretical framework (Campbell and Marshall, 2011; Simpson and Winterheld, 2012), and possibly derives from the scarcity of methods allowing for such a dynamic approach. Although significant insights have been obtained by focusing on individual differences in retrospective reports of the expression of attachment, at present there is scant knowledge regarding how attachment styles are expressed in the moment and how they play out in real-world settings (Torquati and Raffaelli, 2004). The current study extends previous work by employing experience sampling methodology (ESM), a time-sampling procedure, to examine the daily life expression of adult attachment styles in a non-clinical sample of young adults.

Attachment theory is a lifespan approach that postulates that people are born with an innate motivational system (termed the attachment behavioral system) that becomes activated during times of actual or symbolic threat, prompting the individual to seek proximity to particular others with the goal of alleviating distress and obtaining a sense of security (Bowlby, 1982). A cornerstone of the theory is that individuals build cognitive-affective representations, or “internal working models” of the self and others, based on their cumulative history of interactions with attachment figures (Bowlby, 1973; Bartholomew and Horowitz, 1991). These models guide how information from the social world is appraised and play an essential role in the process of affect regulation throughout the lifespan (Kobak and Sceery, 1988; Collins et al., 2004).

The majority of research on adult attachment has centered on attachment styles and their measurement (for a review, see Mikulincer and Shaver, 2007). In broad terms, attachment styles may be conceptualized in terms of security vs. insecurity. Repeated interactions with emotionally accessible and sensitively responsive attachment figures promote the formation of a secure attachment style, characterized by positive internal working models and effective strategies for coping with distress. Conversely, repeated interactions with unresponsive or inconsistent figures result in the risk of developing insecure attachment styles, characterized by negative internal working models of the self and/or others and the use of less optimal affect regulation strategies (Mikulincer and Shaver, 2007).

Although there is a wide range of conceptualizations and measures of attachment insecurity, these are generally defined by high levels of anxiety and/or avoidance in close relationships. Attachment anxiety reflects a desire for closeness and a worry of being rejected by or separated from significant others, whereas attachment avoidance reflects a strong preference for self-reliance, as well as discomfort with closeness and intimacy with others (Brennan et al., 1998; Bifulco and Thomas, 2013). These styles involve distinct secondary attachment strategies for regulating distress – individuals with attachment anxiety tend to use a hyperactivating (or maximizing) strategy, while individuals with attachment avoidance tend to rely on a deactivating (or minimizing) strategy (Cassidy and Kobak, 1988; Main, 1990; Mikulincer and Shaver, 2003, 2008). Indeed, previous empirical studies indicate that attachment anxiety is associated with increased negative emotional responses, heightened detection of threats in the environment, and negative views of the self (Griffin and Bartholomew, 1994; Mikulincer and Orbach, 1995; Fraley et al., 2006; Ein-Dor et al., 2011). By contrast, attachment avoidance is associated with emotional inhibition or suppression, the dismissal of threatening events, and inflation of self-conceptions (Fraley and Shaver, 1997; Gjerde et al., 2004; Mikulincer and Shaver, 2007).

Relatively few studies have examined attachment styles in the context of everyday life. Most of these studies have used event-contingent sampling techniques, such as the Rochester Interaction Record (RIR; Reis and Wheeler, 1991), and have primarily focused on assessing how individual differences in self-reported attachment are related to responses to social interactions in general and/or to specific social interactions (e.g., with acquaintances, friends, family members, close others, same- and opposite-sex peers). Despite various methodological and attachment classification differences that complicate direct comparison of these findings, this body of research has shown that compared to secure attachment, anxious (or preoccupied) attachment is associated with more variability in terms of positive emotions and promotive interactions (a composite measure of disclosure and support; Tidwell et al., 1996), lower self-esteem (Pietromonaco and Barrett, 1997), greater feelings of anxiety and rejection, as well as perceiving more negative emotions in others (Kafetsios and Nezlek, 2002). In contrast, compared to secure attachment, avoidant (or dismissing) attachment has been associated with lower levels of happiness and self-disclosure (Kafetsios and Nezlek, 2002), lower perceived quality of interactions with romantic partners (Sibley and Liu, 2006), a tendency to differentiate less between close and non-close others in terms of disclosure (Pietromonaco and Barrett, 1997), and higher negative affect along with lower positive affect, intimacy, and enjoyment, predominantly in opposite-sex interactions (Tidwell et al., 1996).

Studies using event-contingent methods such as the RIR have shed light on how varying social encounters trigger differential responses as a function of attachment style; however, since the focus is on objectively defined interactional phenomena (e.g., interactions lasting 10 min or longer), these types of paradigms are unable to capture the wide range of naturally occurring subjective states and appraisals that take place as individuals navigate through their daily life. Unlike previous research, the current study used ESM, a within-day self-assessment technique in which participants are prompted at random or predetermined intervals to answer brief questionnaires about their current experiences. ESM offers several advantages compared to traditional laboratory or clinic-based assessment procedures (e.g., deVries, 1992; Hektner et al., 2007; Conner et al., 2009). These include: (1) ESM repeatedly assesses participants in their daily environment, thereby enhancing ecological validity, (2) it captures information at the time of the signal, thus minimizing retrospective recall bias, and (3) it allows for investigating the context of participants’ experiences.

To our knowledge, the work of Torquati and Raffaelli (2004) is the only ESM study that has assessed how daily life experiences of emotion differed as a function of attachment category (secure vs. insecure) and context (being alone or in the presence of familiar intimates). In a sample of undergraduate students, they found that both when in the presence of familiar intimates and when alone, the secure group reported higher levels of emotions relating to energy and connection than the insecure group. Additionally, when alone, securely attached individuals reported greater levels of positive affect than insecurely attached individuals. Moreover, although the two groups did not differ in the variability of their emotional states, participants with a secure style endorsed more extreme positive emotional states across all social contexts, whereas those with insecure styles endorsed more extreme negative emotional states, particularly when they were alone. Their results supported the notion that attachment styles exert a broad influence on affective experiences; nevertheless, an important limitation of this study was that it only reported findings comparing secure vs. insecure participants, and thus it did not provide information on how the subtypes of insecure attachment differ from the secure style. Therefore, further empirical research is needed to examine how attachment styles are expressed in the flow of daily life and whether the interplay between attachment styles and the features of the environment gives rise to different patterns of experiences in the moment. Demonstrating that attachment styles exhibit meaningful associations with real-world experiences in the domains that are theoretically influenced by an individual’s attachment style would provide evidence of the validity of the attachment style construct in the immediate context in which the person is embedded. Moreover, identifying attachment-style variations in how the social context relates to momentary experiences would enhance our understanding of how attachment styles operate in the immediate social milieu.

### The Current Study

The present study examines the expression of secure, anxious, and avoidant attachment styles in daily life using ESM. It extends previous research in several ways. First, the current study employs an interview, rather than a self-report measure, to assess attachment styles. The Attachment Style Interview (ASI; Bifulco et al., 2002) is a semi-structured interview that belongs to the social psychology approach to attachment research and has the strength of utilizing contextualized narrative and objective examples to determine the individual’s current attachment style. Second, this study examines the expression of attachment styles at random time points across participants’ daily life, not just during particular events such as social interactions, and thus captures a more extensive profile of person-environment transactions. Third, this study examines the impact of two aspects of the social context on the expression of attachment styles in the moment: social contact and perceived social closeness when with others. None of the previous diary studies have examined attachment style differences in the effects of social contact and social closeness on participants’ subjective appraisals of themselves (e.g., their coping capabilities), their current situation (e.g., how stressful it is), or their social functioning (e.g., preference for being alone).

The first aim of this study was to examine the associations between attachment styles and measures of affect, cognitive appraisals (about the self, others, and the situation), and social functioning as they occur in daily life. Following attachment theory, it was hypothesized that compared to both insecure attachment groups, secure attachment would be associated with higher ratings of positive affect, self-esteem, feeling cared for, as well as with experiencing more closeness in social interactions. In terms of insecure attachment, a different pattern was predicted for the anxious and avoidant styles. We hypothesized that compared to securely attached participants, those with anxious attachment would endorse higher levels of negative affect, affect instability, subjective stress, feeling unable to cope, and perceived social rejection. We predicted that avoidant attachment, as compared with the secure style, would be associated with lower ratings of positive affect, a decreased desire to be with others when alone, and an increased preference for being alone when with others. In essence, this would provide evidence of ecological construct validity of the attachment styles.

The second aim of the current study was to investigate whether attachment styles moderate the associations of social contact and social closeness with momentary affect, appraisals, and social functioning. Given the lack of engagement and emotional distance that characterizes avoidant attachment, it was hypothesized that social contact would elicit less positive affect in avoidant participants as compared to their secure peers. Additionally, given that one of the most salient features of anxious individuals is that they desire closeness but fear rejection and abandonment, it was predicted that anxious participants would experience higher negative affect with people with whom they did not feel close, than would those with a secure attachment.

## Materials and Methods

### Participants

Participants were 206 (44 men, 162 women) undergraduate students recruited from the Universitat Autònoma de Barcelona in Spain. The mean age of the sample was 21.3 years (*SD* = 2.4). An additional eight participants enrolled in the study and completed the interview phase, but were omitted from the analyses due to failing to complete the ESM protocols. Ethical approval for the study was obtained from the University Ethics Committee. Participants provided written informed consent and were paid for their participation.

### Materials and Procedure

Participants were assessed with the ASI, along with other interview and questionnaire measures not used in the present study. The ASI is a semi-structured interview that measures current attachment style through questions that elicit the content and context of interpersonal attitudes and behaviors (Bifulco, 2002). The interview is composed of two parts. In the first part, a behavioral evaluation of the ability to make and maintain relationships is made (on a 4-point scale from “marked” to “little/none”) on the basis of the overall quality of the person’s ongoing relationships with up to three supportive figures (referred to as “very close others”), including partner if applicable. The term “behavioral evaluation” denotes that ratings are based on descriptions of actual behavior (such as instances of recent confiding, emotional support received, and presence of tension/conflicts with each “very close other”). The second part of the ASI assesses individuals’ feelings and thoughts about themselves in relation to others. Specifically, ratings are obtained for seven attitudinal scales that reflect anxiety and avoidance in relationships. These scales are: fear of rejection, fear of separation, desire for company, mistrust, anger, self-reliance, and constraints on closeness. Ratings on the attitudinal scales are based on the intensity of the attitude and the level of generalization. Most of them are rated on 4-point scales from “marked” to “little/none.”

The scores obtained throughout the interview are combined to enable the classification of the person’s attachment profile, which encompasses both the attachment style categorization as well as the degree of severity for the insecure styles. Note that scoring the ASI and deriving the person’s attachment profile is done on the basis of prior training, according to established rating rules and benchmark thresholds. Further details on the scoring scheme and case examples can be found in Bifulco and Thomas (2013). Previous studies have provided evidence for the reliability and validity of the ASI (Bifulco et al., 2004; Bifulco and Thomas, 2013). In the present study, the three main attachment style categories (i.e., secure, anxious, and avoidant) were used for analyses.

Experience sampling methodology data were collected on palm pilot personal digital assistants (PDAs). The PDAs signaled the participants randomly eight times a day (between 10 a.m. and 10 p.m.) for 1 week to complete brief questionnaires. When prompted by the signal, the participants had 5 min to initiate responding. After this time window or upon completion of the questionnaire, the PDA would become inactive until the next signal. Each questionnaire took ∼2 min to complete.

The ESM questionnaire included items that inquired about the following domains: (1) affect in the moment, (2) appraisals about the self, (3) appraisals about others, (4) appraisals of the current situation, (5) social contact, and (6) social appraisals and functioning (see **Table 1** for the English translation of the ESM items used in the present study). The social contact item (i.e., “Right now I am alone”) was answered dichotomously (yes/no), whereas the remaining items were answered using 7-point scales from 1 (not at all) to 7 (very much). Note that for the sake of aiding the interpretation of the results we have made a distinction between affective states and cognitive appraisals; however, we recognize that such a distinction is not clear-cut and that affect and cognition are complexly intertwined processes. Likewise, we grouped appraisals as pertaining to the self, others, or the situation. This distinction is somewhat artificial but useful for organizing the presentation of the data. Note that, unlike most previous studies, the label “appraisals about others” does not refer to participants’ ratings of interaction partners, but to the manner in which participants’ experience others’ motives, actions, or esteem toward them.

**Table 1:** Direct effects of attachment style on daily life experiences.

Level 1 criterion	Level 2 predictors	
	Anxious vs. Secure γ_01_ (*df* = 203)	Avoidant vs. Secure γ_02_ (*df* = 203)
**Affect in the moment**		
Right now I feel happy	-0.526 (SE = 0.148)^∗∗∗^	-0.426 (SE = 0.147)^∗∗^
Right now I feel relaxed	-0.483 (SE = 0.150)^∗∗^	-0.151 (SE = 0.144)
Right now I fear losing control	1.032 (SE = 0.289)^∗∗∗^	0.091 (SE = 0.371)
Negative affect index	0.341 (SE = 0.089)^∗∗∗^	0.065 (SE = 0.103)
**Appraisals about the self**		
Right now I feel good about myself	-0.695 (SE = 0.149)^∗∗∗^	-0.384 (SE = 0.163)^∗^
Right now I feel guilty or ashamed	0.266 (SE = 0.076)^∗∗^	0.214 (SE = 0.109)^∗^
Right now I can cope	-0.591 (SE = 0.143)^∗∗∗^	-0.368 (SE = 0.160)^∗^
**Appraisals about others**		
Right now I feel that others care about me	-0.439 (SE = 0.194)^∗^	-0.520 (SE = 0.212)^∗^
Right now I feel suspicious	0.314 (SE = 0.087)^∗∗∗^	0.083 (SE = 0.064)
Right now I feel mistreated	1.030 (SE = 0.317)^∗∗^	0.752 (SE = 0.384)
**Appraisals about the situation**		
I like what I’m doing right now	-0.398 (SE = 0.142)^∗∗^	-0.231 (SE = 0.121)
Right now I can do my current activity	-0.377 (SE = 0.135)^∗∗^	-0.127 (SE = 0.146)
My current situation is positive	-0.687 (SE = 0.177)^∗∗∗^	-0.402 (SE = 0.158)^∗^
My current situation is stressful	0.560 (SE = 0.185)^∗∗^	0.058 (SE = 0.174)
**Social appraisals and functioning**		
Right now I am alone	0.025 (SE = 0.129)	-0.242 (SE = 0.152)
When alone:		
I am alone because people do not want to be with me	1.288 (SE = 0.501)^∗^	0.245 (SE = 0.560)
Right now I would prefer to be with people	-0.018 (SE = 0.219)	-0.435 (SE = 0.210)^∗^
When with others:		
I feel close to this person (these people)	-0.434 (SE = 0.146)^∗∗^	-0.379 (SE = 0.158)^∗^
Right now I would prefer to be alone	0.488 (SE = 0.126)^∗∗∗^	0.373 (SE = 0.134)^∗∗^

Negative affect index was computed by averaging the scores for the following three items: “Right now I feel sad,” “Right now I feel anxious,” and “Right now I feel angry.” ^∗^*p* < 0.05, ^∗∗^*p* < 0.01, ^∗∗∗^*p* < 0.001.

### Statistical Method

Experience sampling methodology data have a hierarchical structure in which daily life ratings (level 1 data) are nested within participants (level 2 data). Multilevel or hierarchical linear modeling techniques are a standard approach for the analysis of ESM data (Nezlek, 2001; Bolger and Laurenceau, 2013). The multilevel analyses examined two types of relations between the attachment groups and daily life experiences. First, we assessed the independent effects of level 2 predictors (attachment style groups) on level 1 dependent measures (ESM ratings in daily life). Second, cross-level interactions (or slopes-as-outcomes) examined whether level 1 relationships (e.g., closeness and negative affect in the moment) varied as a function of level 2 variables (attachment groups). The analyses were conducted with Mplus 6 (Muthén and Muthén, 1998–2010). To examine the effects of attachment, the analyses included two dummy-coded attachment style variables that were entered simultaneously as the level 2 predictors, following Cohen et al. (2003). The first dummy code contrasted the anxious and secure attachment groups, and the second contrasted the avoidant and secure attachment groups. The secure attachment group was coded 0 in both codings. Note that direct comparisons of the anxious and avoidant attachment groups were not made, given that our hypotheses focused on differences between secure and insecure attachment. Level 1 predictors were group-mean centered (Enders and Tofighi, 2007). The data departed from normality in some cases, so parameter estimates were calculated using maximum likelihood estimation with robust SEs.

## Results

Based upon the ASI, 119 (57.8%) of the participants were categorized as having secure attachment, 46 (22.3%) as having anxious attachment, and 41 (19.9%) as having avoidant attachment. These percentages are comparable to those reported in previous studies using the ASI in non-clinical samples (e.g., Conde et al., 2011; Oskis et al., 2013). The attachment groups did not differ in terms of age or sex. Participants completed an average of 40.8 usable ESM questionnaires (*SD* = 9.1). The attachment groups did not differ on the mean number of usable questionnaires (Secure = 40.8, *SD* = 8.2; Anxious = 40.5, *SD* = 9.8; Avoidant = 41.1, *SD* = 10.9).

### Expression of Attachment Styles in Daily Life

**Table 1** presents the direct effects of attachment on daily life experiences. Compared to participants with a secure attachment, those with an anxious attachment reported higher negative affect, lower positive affect, as well as greater fear of losing control in daily life. As expected, the avoidant and secure groups did not differ in their ratings of negative affect, but avoidant participants reported feeling less happy than their secure counterparts. In addition to comparing the attachment groups on the experience of mean levels of affect in daily life, we also compared the groups on variance of affect using one-way ANOVAs. Note that this was not nested data because each participant had a single (within-person) variance score based upon their own distribution of happiness or negative affect. The ANOVA was significant for negative affect variance, *F*(2,203) = 5.58, *p* < 0.01. *Post-hoc* comparisons using Dunnett’s *t*-test indicated that the anxious attachment group exceeded the secure attachment group, *p* < 0.01. The avoidant and secure attachment groups did not differ. The ANOVA for happiness variance was not significant, *F*(2,203) = 0.48.

The attachment styles were also differentiated by their appraisals of the self, others, and the situation. Relative to both insecure groups, secure individuals endorsed more positive views on all items tapping appraisals about the self. That is, both anxious and avoidant participants perceived themselves in a more negative manner and were less confident in their coping capacities. Consistent with our hypotheses, individuals with an anxious or avoidant style reported feeling less cared for by others than did those with a secure attachment. Participants with an anxious style also differed from their secure peers in that they felt more suspicious and mistreated in the moment. In terms of appraisals about the situation, compared to secure attachment, anxious attachment was associated with expressing decreased enjoyment and competence regarding current activities, as well as with reports that the current situation was less positive and more stressful. Avoidant participants perceived their immediate situation as less positive, but not as more stressful, than secure participants.

Regarding social appraisals and functioning, the attachment groups did not differ in terms of how often they were with other people at the time of the signal (on average, secure participants were alone 42.6% of the time, anxious participants 41.9% of the time, and avoidant participants 48.1% of the time). Participants with a secure style reported greater feelings of closeness than did those with an anxious or avoidant style. As expected, anxiously attached individuals were more likely than secure ones to report that they were alone because others did not want to be with them (i.e., perceived social rejection). Moreover, as compared with secure individuals, those with an avoidant attachment showed a decreased desire to be with others when alone, and an increased preference to be alone when with others. Unexpectedly, compared with the secure group, the anxious group also displayed a higher preference for being alone when with others.

### Moderating Effects of Attachment Style on the Association of Social Context with Daily Life Experiences

Two sets of cross-level interaction analyses were conducted to examine the extent to which participants’ social context impacted the expression of attachment styles in daily life. Specifically, we examined whether attachment styles moderated the association of social contact (alone = 1; with others = 2) and social closeness when with others (“I feel close to this person [people]”; ranging from 1 to 7) with measures of affect, appraisals, and functioning in the moment (**Table 2**). Overall, the report of being with other people at the time of the signal was significantly associated with experiencing greater happiness, decreased negative affect, having more positive self-appraisals, feeling more cared for by others, as well as with viewing one’s situation more positively. However, these associations were not moderated by attachment style, indicating that the impact of social contact on daily life experiences was not differentially expressed for the attachment groups.

**Table 2:** Cross-level interactions of social contact and social closeness with daily life experiences.

Level 1 criterion	Level 1 predictor		Level 2 predictors^@^	
	γ_10_ (*df* = 203)		Anxious vs. Secure γ_11_ (*df* = 203)	Avoidant vs. Secure γ_12_ (*df* = 203)
Right now I feel happy	Contact	0.393 (0.035)^∗∗∗^	0.001 (0.090)	-0.002 (0.090)
Negative affect index	Contact	-0.049 (0.021)^∗^	-0.033 (0.058)	0.030 (0.048)
Right now I feel that others care about me	Contact	0.403 (0.042)^∗∗∗^	-0.120 (0.098)	0.154 (0.121)
Right now I feel good about myself	Contact	0.174 (0.026)^∗∗∗^	-0.034 (0.067)	-0.019 (0.065)
Right now I can cope	Contact	0.143 (0.029)^∗∗∗^	-0.027 (0.084)	0.030 (0.069)
My current situation is positive	Contact	0.245 (0.027)^∗∗∗^	-0.060 (0.072)	0.007 (0.065)
My current situation is stressful	Contact	0.010 (0.038)	0.027 (0.101)	0.192 (0.106)
Right now I feel happy	Closeness	0.161 (0.014)^∗∗∗^	0.068 (0.032)^∗^	0.012 (0.036)
Negative affect index	Closeness	-0.059 (0.010)^∗∗∗^	-0.076 (0.026)^∗∗^	-0.006 (0.023)
Right now I feel that others care about me	Closeness	0.144 (0.016)^∗∗∗^	0.028 (0.038)	0.094 (0.046)^∗^
Right now I feel good about myself	Closeness	0.072 (0.013)^∗∗∗^	0.045 (0.029)	0.051 (0.035)
Right now I can cope	Closeness	0.061 (0.013)^∗∗∗^	0.095 (0.038)^∗^	-0.002 (0.032)
Right now prefer to be alone	Closeness	-0.268 (0.020)^∗∗∗^	-0.118 (0.050)^∗^	-0.084 (0.058)
My current situation is positive	Closeness	0.120 (0.014)^∗∗∗^	0.127 (0.040)^∗∗^	0.050 (0.037)
My current situation is stressful	Closeness	-0.139 (0.017)^∗∗∗^	-0.123 (0.047)^∗∗^	-0.029 (0.050)

^@^Cross-level interaction of the association of the attachment groups with the slope of the level 1 predictor and criterion ^∗^*p* < 0.05 ^∗∗^*p* < 0.01 ^∗∗∗^*p* < 0.001.

**FIGURE 1:**
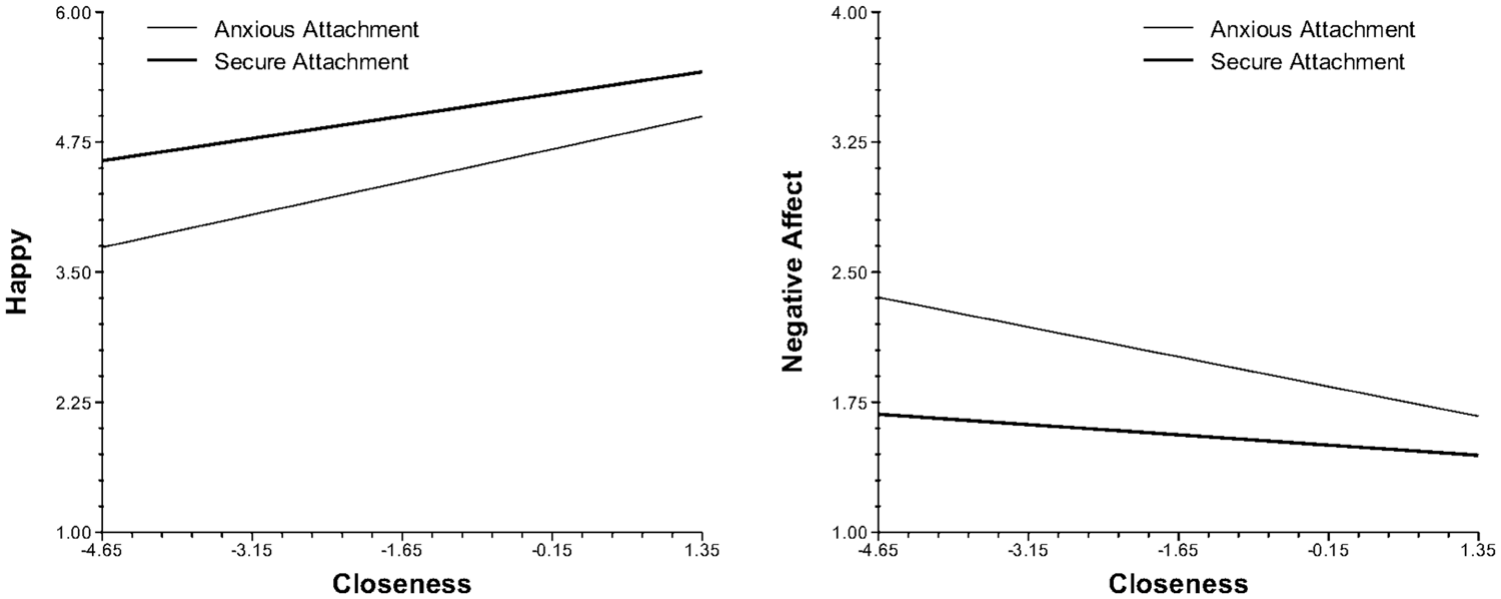
Cross-level interaction of attachment style with social closeness and affective experiences in daily life.

**FIGURE 2:**
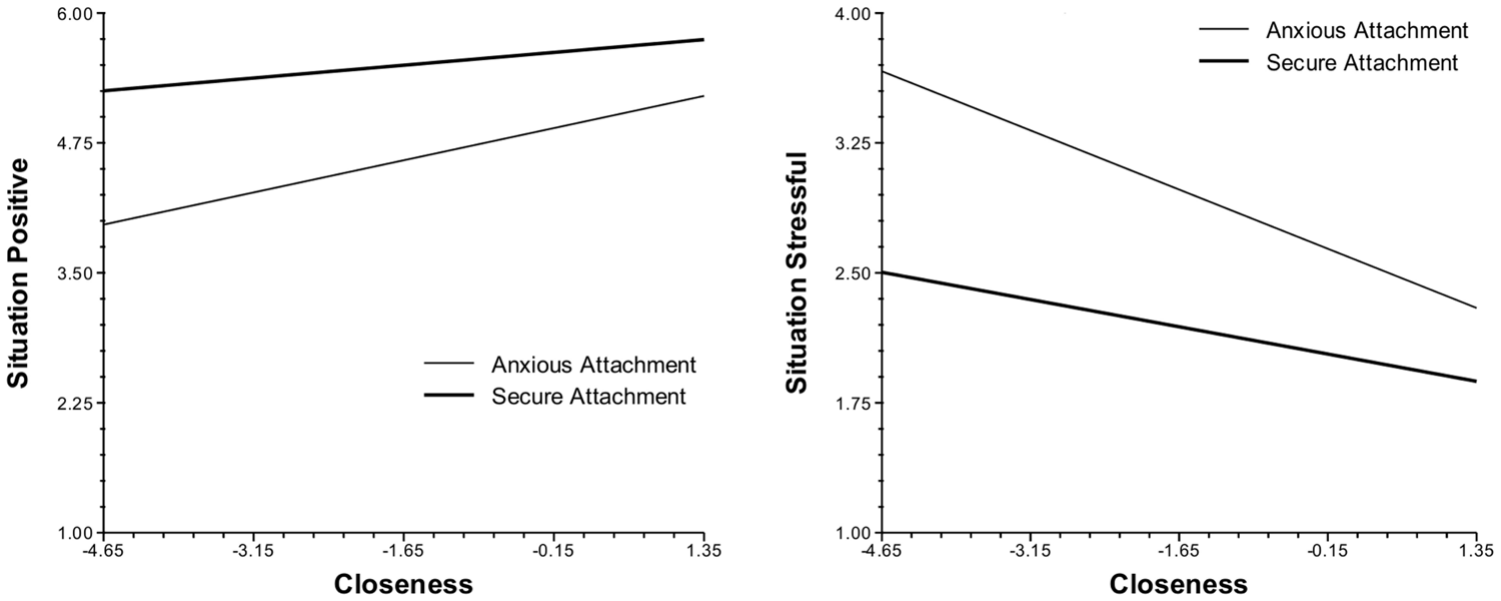
Cross-level interaction of attachment style with social closeness and situation appraisals in daily life.

**FIGURE 3:**
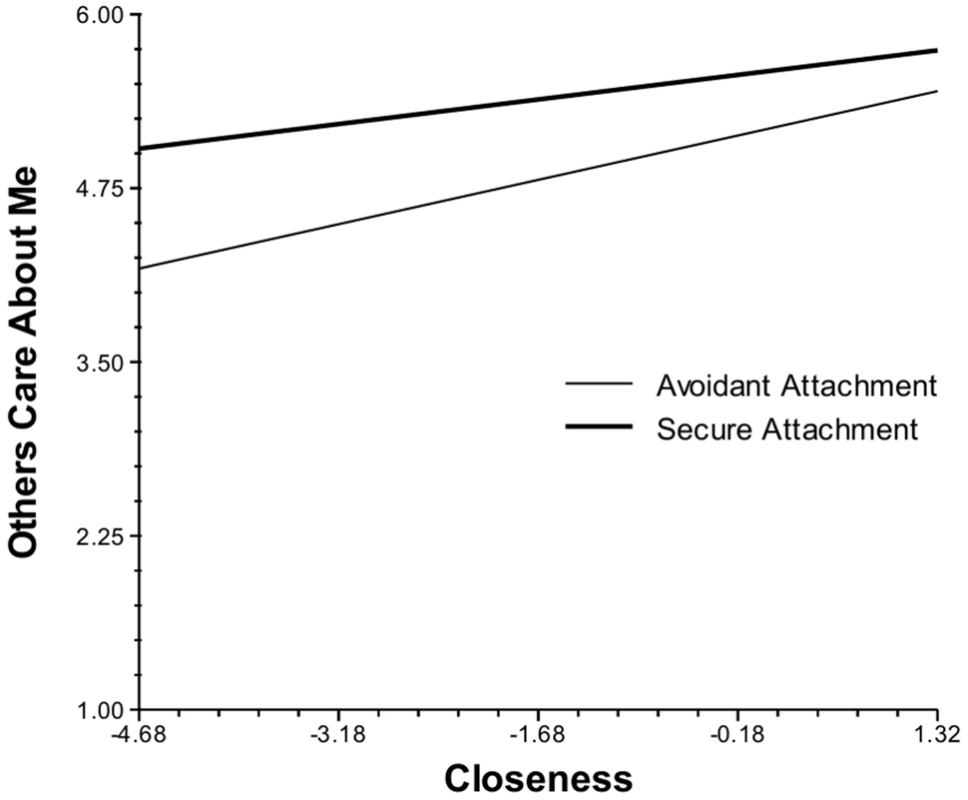
Cross-level interaction of attachment style with social closeness and feeling cared for by others in daily life.

The closeness of social contacts in the moment was also associated with the momentary experience of affect, appraisals, and functioning. However, in contrast to social contact, the effects of social closeness on daily life experiences were significantly moderated by attachment style. When in the presence of people they did not feel close to, anxious participants reported more negative and less positive experiences than did those with a secure attachment. Specifically, as closeness diminished, anxious individuals experienced greater decreases in happiness and increased negative affect (**Figure 1**), appraised their current situation as less positive and more stressful (**Figure 2**), experienced greater decreases in their ability to cope, and reported a stronger preference for being alone than their securely attached peers. Cross-level analyses also revealed that as closeness diminished, avoidant participants felt less cared for by others than did those with a secure attachment (**Figure 3**).

## Discussion

To our knowledge, the current study is the first to examine how adult attachment styles, as measured by interview, are expressed in daily life using ESM in a sample of non-clinical young adults. As hypothesized, we found that participants’ momentary affective states, cognitive appraisals, and social functioning varied in meaningful ways as a function of their attachment style. These results support the construct and ecological validity of the ASI as a sensitive measure of attachment styles. Furthermore, they extend previous research by demonstrating that the effects of attachment style on daily life experiences are manifested across a variety of contexts and are not limited to interactional settings. In addition, the present study investigated the impact of the social context on the expression of attachment styles in the moment. The findings indicated that insecure individuals are especially reactive to the subjective nature of social contacts in their everyday life, not simply to the impact of whether they are alone or with others.

### Attachment Strategies in Daily Life

Overall, the results regarding the daily life expression of attachment styles confirmed our theory-based predictions. Relative to both anxious and avoidant participants, those holding a secure style reported greater feelings of happiness, more positive self-appraisals, viewed their current situation more positively, felt more cared for by others, and felt closer to the people they were with. These findings are consistent with previous work showing that secure attachment is associated with a sense of self-efficacy, optimistic appraisals toward life in general, as well as positive interpersonal attitudes (Mikulincer and Shaver, 2007, 2008). Moreover, the pattern of positive momentary experiences reported by secure, as compared to insecure, participants supports the notion that attachment security allows individuals to engage with their environment in a way that fosters psychological and relational benefits (Siegel, 2012).

In the present study, the most pronounced differences emerged between the secure and anxious attachment groups. These differences showed that the daily experiences of individuals with an anxious style were consistent with the use of hyperactivating strategies. That is, compared with their secure peers, anxious participants approached their daily person-environment transactions with amplification of distress (e.g., higher negative affect, greater fear of losing control, higher subjective stress), decreased positive affect, and greater variability in the experience of negative affect. These results support Mikulincer and Shaver’s (2003, p. 109) characterization of anxiously attached people as possessing a “chaotic emotional architecture” that contributes to the dysregulation of negative affect. We also found that anxiously attached participants endorsed more negative and less positive appraisals about themselves and their current situation than their secure counterparts, which supports the negative effects of hyperactivating strategies on people’s cognitive appraisals. Moreover, relative to secure participants, anxious ones felt less cared for by others, less close to the people they were with, more suspicious, more mistreated, and, when alone, were more likely to hold attributions of not being wanted. This pattern of findings provides strong empirical evidence that the appraisals that anxious individuals make in the realm of daily life are characterized by a hypervigilance to interpersonal sources of threat and hypersensitivity toward rejection. The results also revealed that when anxiously attached participants were with others, they displayed a stronger preference for being alone than their secure peers. Although this finding was not expected, the cross-level interactions seem to suggest that this is driven by a heightened discomfort that arises when anxious individuals are in the presence of people with whom they do not feel close.

In regards to avoidantly attached participants, the results showed that their daily life experiences were consistent with the reliance on deactivating strategies. As predicted, compared with secure subjects, avoidant ones endorsed a stronger preference for being alone when with others and a decreased desire to be with others when alone. Additionally, relative to their secure peers, they tended to approach their person-environment transactions with decreased happiness and less positive views of their situation, but not with amplification of negative states. Avoidant participants also felt less cared for by others and less close to the people they were with than did secure participants. This is consistent with their psychological barriers toward closeness and possibly indicates that their lack of involvement in relationships that elicit closeness and care may reinforce their underlying models in a self-perpetuating manner. Avoidant individuals also reported more negative views of themselves than did those with a secure attachment. Although avoidantly attached people have often been conceptualized as holding a positive self-model (Bartholomew and Horowitz, 1991), research suggests that their positive views of themselves reflect defensive processes of self-inflation (Mikulincer and Shaver, 2007). It could be that when asked to report on their experiences in the moment, avoidant individuals are less capable of suppressing the vulnerable nature of their sense of self. Indeed, it has been posited that ESM assessments allow less room for people to resort to self-interpretation or use mental heuristics when reporting on their self-perceptions (Delespaul, 1995).

### The Impact of Social Context on the Expression of Attachment Styles

Contrary to our initial expectation, the impact of social context on the expression of attachment styles in the moment was only observed for social closeness and not for social contact. This finding is important because it highlights a boundary condition of the effects of attachment style in social contexts — namely, that the manifestation of attachment styles depends on the subjective appraisal of the closeness of social contacts, rather than on the simple presence of social interactions. The finding that it is social appraisals, not simply social contact, that interacts with attachment is compatible with the description of attachment as a “person by situation” interactionist theory that at its core involves appraisal of the social context.

Increased levels of perceived closeness were associated with differential responses for anxious and avoidant individuals. Compared with the secure group, the affective states, situation appraisals, coping capacities, and social functioning of the anxious group worsened as closeness diminished; or, seen from the opposite perspective, improved as closeness increased. This pattern of results may be interpreted to suggest that when in the presence of people they do not feel close to, anxious people’s preoccupation with rejection and approval is amplified and this permeates their subjective experiences. By contrast, increased levels of closeness might enhance their momentary sense of felt-security and provide them with the self-validation they long for, which in turn could bring about an improvement in their subjective experiences. The finding that greater closeness seemed to aid anxious participants with the regulation of various self-states (e.g., affect, coping, stress) resonates with the work of Pietromonaco and Barrett (2006), who, using a variant of the RIR, concluded that individuals holding a preoccupied attachment valued their interacting partners more when the interactions had provided help with self-regulatory processess.

The results also demonstrated that as closeness diminished avoidant subjects felt less cared for by others than their secure peers. Because avoidant individuals approach their interpersonal interactions in a way that minimizes the possibility of frustration (in order to keep their attachment system deactivated), it may be that experiencing closeness disconfirms their low expectations (e.g., about others’ responsiveness) and thus makes them more perceptive to the caring attitudes of others. Notably, the fact that greater closeness affected appraisal about others, but not their self-states, is in line with the contention that avoidantly attached people resort to autoregulation (i.e., they turn to themselves to regulate their internal states; Solomon and Tatkin, 2011). Additional research is required to elucidate the specific psychological mechanisms that make up the experience of momentary closeness and how it is associated with beneficial effects for insecurely attached individuals.

### Specificity of Attachment Processes in Daily Life

The results of this study are relevant to the broader debate in the attachment field regarding the specificity of attachment-related processes in adulthood (see Tidwell et al., 1996; Pietromonaco and Barrett, 1997; Torquati and Raffaelli, 2004). On the one hand, the fact that attachment styles predicted individual’s subjective experiences across the range of situations they encountered during the week, and not only those that were interaction-based, suggests that attachment styles are relevant features of personality functioning that have pervasive effects on how individuals experience their inner and outer worlds. On the other hand, the findings that attachment styles moderated the effects of perceived social closeness on daily life experiences (but not the effects of mere social contact on these experiences) highlights the fact that attachment styles are differentially expressed under relational circumstances that might bring attachment concerns to the fore. Thus, we believe that a richer understanding of attachment dynamics will come from efforts that examine their expression at both the individual and relational level.

### Limitations and Future Directions

Additional research is warranted to address the limitations of the present study. First, we used a sample of college students with predominantly female participants. Future studies would benefit from assessing the expression of attachment styles in community samples with a wider age range and a more representative distribution in terms of gender. Second, it should be noted that the cross-level interactions of the effects of social closeness on the expression of attachment styles were interpreted in line with theoretical propositions from the attachment literature; nevertheless, given the correlational nature of these data, the opposite interpretation is also plausible (e.g., less coping capacity contributing to lower perceived closeness). Third, note that the attachment groups showed a broader pattern of significant results on the direct effects than the interactions. This likely demonstrates the robust nature of the direct effects and the fact that the interactions are computed over-and-above the direct effects. Thus, we want to be careful not to over-interpret the cross-level interaction effects. Nevertheless, we believe that the pattern of findings for the cross-level interactions indicates that anxious attachment (relative to secure attachment) is reactive to the nature of social contact, not simply any social contact; whereas avoidant attachment generally is not characterized by strong reactivity to social context (as measured in the current study). Fourth, this study focused exclusively on momentary appraisals of social closeness. Further research could expand upon the current findings by assessing the effects of variations in trait social closeness (e.g., Moore et al., 2014). Finally, it would also be important for future work to assess the extent to which our findings are generalizable across different cultures. Given that we found theoretically expected daily life correlates of attachment styles in a Spanish sample, the results would seem to fit with the notion that attachment strategies are universal characteristics (van IJzendoorn and Sagi-Schwartz, 2008; van IJzendoorn and Bakermans-Kranenburg, 2010). However, studies in different cultures are needed to establish the cross-cultural ecological validity of attachment styles.

## Conclusion

The extent to which attachment style differences are expressed in real time as individuals navigate their real-life settings has remained an area largely untapped by research in the attachment field. The present investigation provided a novel contribution by using an interview-based measure to assess adult attachment styles and by employing a random time-sampling procedure that demonstrated that the hallmark features of secure, anxious, and avoidant individuals are reflected in their day-to-day person-environment transactions. The current study further extends the validity of the attachment style construct to the realm of everyday life and, moreover, points to the utility of employing ESM for obtaining a more finely grained understanding of how the predictions derived from attachment theory play out in the natural flow of real life.

## Author Contributions

TS contributed to study design, data collection, data management, and writing of the manuscript. TK contributed to study conception, study design, data analyses, and writing of the manuscript. SB contributed to data collection and critically revised the manuscript. MM contributed to data collection and critically revised the manuscript. CC contributed to data analyses and critically revised the manuscript. PS contributed to study design, provided input regarding data analyses, and critically revised the manuscript. NB-V was the principal investigator, conceived the study and contributed to study design, data collection, and writing of the manuscript. All authors have read and approved the final manuscript.

## Conflict of Interest Statement

The authors declare that the research was conducted in the absence of any commercial or financial relationships that could be construed as a potential conflict of interest.
